# Job satisfaction among healthcare providers in Saudi tertiary government hospitals: findings from a cross-sectional study

**DOI:** 10.3389/fpsyg.2025.1651861

**Published:** 2026-01-20

**Authors:** Nawal A. Alissa, Nouf Qatnan Alotaibi

**Affiliations:** 1 Department of Community Health Sciences, College of Applied Medical Sciences, King Saud University, Riyadh, Saudi Arabia; 2 Dental Center, Prince Sultan Military Medical City, Riyadh, Saudi Arabia

**Keywords:** job satisfaction, healthcare providers, job satisfaction scale, government hospitals, Saudi Arabia

## Abstract

**Background:**

Job satisfaction is a key determinant of healthcare provider performance and well-being, influencing both organizational effectiveness and the quality of patient care. In tertiary care settings, nursing and support staff often face high workloads and complex demands that may affect satisfaction levels. This study aimed to assess job satisfaction among healthcare providers in two tertiary government hospitals in Riyadh, Saudi Arabia, and to examine differences across professional roles and satisfaction domains.

**Methods:**

A cross-sectional study was conducted among 135 healthcare providers at King Khalid University Hospital and Prince Sultan Military Medical City (February–March 2025). Job satisfaction was assessed using a validated 36-item structured questionnaire adapted from Deshmukh et al. (2023), covering nine core domains. Descriptive statistics summarized participant characteristics and satisfaction scores. Chi-square tests examined associations between categorical variables and satisfaction levels, independent samples *t*-tests compared mean scores between two groups, and one-way ANOVA assessed differences across multiple groups. A *p*-value <0.05 was considered statistically significant.

**Results:**

Physicians reported the highest mean job satisfaction (173.4 ± 21.2), followed by nurses (169.8 ± 18.7) and support staff (165.2 ± 20.5). The highest satisfaction was observed in the 31–40-year age group (84.5%). Fringe benefits (mean = 19.96) and contingent rewards (17.23 ± 3.87) were the top-rated satisfaction domains. Nurses demonstrated moderate satisfaction, identifying areas for managerial attention.

**Conclusion:**

The study demonstrates variations in job satisfaction across healthcare provider roles and domains, indicating that satisfaction is influenced by both professional and demographic factors. These findings provide a basis for future organizational strategies aimed at improving healthcare workforce well-being.

## 1 Introduction

Job satisfaction is a fundamental determinant of employee performance and organizational effectiveness, particularly within the healthcare sector. It is commonly defined as an employee’s overall evaluation of their work experience, reflecting the degree to which their job meets psychological, professional, and organizational expectations (Bai et al., 2021). Healthcare providers, including clinicians, nurses, allied health professionals, and medical educators, frequently operate in high pressure environments where job satisfaction directly influences their psychological wellbeing and the quality of care they deliver. In academic tertiary hospitals, where clinical service is integrated with teaching and training, the satisfaction of healthcare professionals also impacts the learning environment and the quality of medical education. Research consistently demonstrates that satisfied healthcare workers exhibit higher levels of motivation, productivity, and commitment, thereby enhancing both patient outcomes and the effectiveness of educational programs (Gedif et al., 2018). In contrast, dissatisfaction contributes to burnout, absenteeism, and high turnover rates, all of which compromise care delivery, team continuity, and the mentoring of future health professionals (Bai et al., 2021).

The global healthcare sector is confronting escalating challenges, including rising patient demands, critical workforce shortages, and constrained resources, all of which place increasing pressure on healthcare systems and professionals. These challenges are intensified in tertiary care hospitals, where professionals manage complex cases that require advanced skills, long working hours, and, in many instances, contribute to clinical teaching. Ensuring high job satisfaction in such demanding environments is therefore critical to sustaining a resilient workforce capable of delivering both clinical excellence and educational leadership (Bai et al., 2021).

Saudi Arabia’s healthcare system has undergone major reforms guided by Vision 2030, which seeks to enhance healthcare accessibility, quality, and sustainability. Achieving these ambitious goals depends not only on policy and infrastructure development but also on the satisfaction and motivation of healthcare providers, whose performance directly influences care quality, workforce stability, and the success of educational reforms. Tertiary care government hospitals play a key role in these efforts by providing advanced medical services and supporting medical education through clinical teaching and the supervision of interns, residents, and students (Almalki et al., 2021). These institutions accommodate high patient volumes while simultaneously serving as benchmarks for clinical training and national healthcare strategies (Alotaibi et al., 2022).

Riyadh, the capital of Saudi Arabia and a key center for medical education and healthcare innovation, hosts several tertiary government hospitals staffed by a diverse workforce comprising both Saudi and expatriate professionals (Alotaibi et al., 2022). These healthcare providers often contend with significant challenges, including high workload demands, cross-cultural communication barriers, and administrative inefficiencies, all of which may adversely affect their job satisfaction. When medical educators are among those affected, diminished satisfaction can also compromise the quality of clinical instruction and mentorship offered to medical students and trainees (Al Bazroun et al., 2023; Albugami et al., 2024).

Job satisfaction among healthcare professionals is multifaceted, encompassing emotional, psychological, and practical dimensions that influence both individual performance and systemic efficiency (Bai et al., 2021; Ramli, 2019). At its core, job satisfaction reflects the extent to which individuals feel content and motivated in their roles. Burnout and emotional exhaustion, particularly in tertiary settings, have been linked to high workload and limited support (Alghamdi and Alotaibi, 2023). In Saudi Arabia, cultural expectations and hierarchical systems may further complicate the experience of healthcare professionals, particularly expatriates, who may face disparities in compensation, recognition, and career development opportunities (Tariq et al., 2021; Yousef, 2022).

Leadership and institutional culture also shape how satisfied employees feel. Transformational leadership, which is characterized by support, communication, and recognition, is associated with higher satisfaction levels (Notarnicola et al., 2024). Conversely, rigid or transactional leadership approaches may fail to meet the emotional and professional needs of healthcare staff, contributing to disengagement and dissatisfaction (Al-Dosari et al., 2023).

Maintaining work life balance is essential for job satisfaction. Research has shown that factors such as shift length, overtime, and inadequate rest periods can significantly impact satisfaction levels (Almalki et al., 2012; Senek et al., 2020). Healthcare workers, especially in high intensity settings like tertiary hospitals, benefit from institutional policies that promote fair workload distribution and flexible scheduling (Al-Dubai et al., 2020).

Recognition systems and access to professional growth opportunities also contribute to job satisfaction. In line with Vision 2030, many government hospitals are investing in staff development initiatives. These include continuing education, structured career pathways, and opportunities for involvement in decision making, all of which are associated with improved job satisfaction and retention (Alghamdi et al., 2021).

Teamwork and collaboration are fundamental in healthcare environments. The interdisciplinary nature of tertiary hospitals requires effective communication and mutual respect across professional roles (Raoush, 2023). When these elements are lacking, staff may experience role ambiguity and interpersonal conflict, both of which are detrimental to job satisfaction (Sadeghi et al., 2021).

While global research consistently identifies key factors that influence job satisfaction such as workload, salary, interpersonal relationships, leadership style, and opportunities for career development, these elements must be understood within the cultural and administrative context of the Saudi healthcare system. Despite the recognized importance of job satisfaction in promoting workforce stability and healthcare quality, there is a noticeable lack of comprehensive research within tertiary government hospitals in Riyadh, particularly those that combine clinical service with medical education. This gap limits the ability of healthcare leaders and decision makers to develop strategies that effectively address the needs of healthcare professionals.

Factors including organizational culture, fair compensation, work life balance, and access to professional resources require more focused examination to inform targeted improvements (Asegid et al., 2014). Supporting job satisfaction is not only important for retaining qualified staff and promoting their wellbeing, but also plays a critical role in ensuring high quality patient care and sustaining effective clinical education. As Saudi Arabia advances its healthcare transformation under Vision 2030, giving priority to healthcare provider satisfaction will be essential to achieving meaningful and lasting progress (Assiri et al., 2020).

This study aims to address this gap by assessing the level of job satisfaction and identifying its key influencing factors among a broad range of healthcare professionals working in tertiary care institutions in Riyadh, Saudi Arabia. In contrast to previous studies that often concentrate on single professional groups or general hospital settings, this research includes doctors, nurses, and allied health staff to provide a more inclusive and context specific understanding of satisfaction. Conducted during a time of national reform, the study offers timely evidence to guide institutional practices, support staff wellbeing, and inform strategic planning within Saudi Arabia’s evolving healthcare landscape.

## 2 Methods

### 2.1 Study design

This study aimed to assess job satisfaction among healthcare providers in two tertiary government hospitals in Riyadh, Saudi Arabia, and to examine differences across professional roles and satisfaction domains.

### 2.2 Study setting

The study was carried out at King Khalid University Hospital (KKUH) and Prince Sultan Military Medical City (PSMMC), both located in Riyadh, Saudi Arabia. KKUH is a multidisciplinary teaching hospital affiliated with King Saud University. It provides primary, secondary, and tertiary healthcare services with a capacity of 800 beds and a workforce of over 4,226 employees. As a leading academic and clinical institution, KKUH plays a pivotal role in delivering healthcare services, supporting medical education, and advancing health research (Medical City King Saud University, 2019).

PSMMC, established in 1997 under the Ministry of Defense, is one of the largest military medical complexes in Saudi Arabia. It has expanded from 385 to 920 beds and employs approximately 4,810 staff. PSMMC serves military personnel, their dependents, and civilian patients, delivering a wide range of specialized medical services (Prince Sultan Military Medical City, 2022).

These two hospitals were selected due to their national prominence, diverse healthcare workforces, and dual roles in patient care and clinical training. They serve a broad patient population and represent critical institutions within the Saudi healthcare system, making them appropriate sites for exploring job satisfaction among healthcare providers.

### 2.3 Study population

The target population comprised healthcare providers (including physicians, nurses, and allied health professionals) working at KKUH and PSMMC during the academic year 1446/2025. Participation was voluntary, and informed consent was obtained from all respondents.

### 2.4 Sample size and sampling technique

From a total workforce of 9,036 healthcare employees across both hospitals, a representative sample of 369 participants was selected using a simple random sampling technique. The sample size was calculated using the following formula:


$$n_{0}=\frac{Z^{2}\cdot p\cdot (1-p)}{e^{2}}$$

where

- *Z* = 1.96 (for 95% confidence level)
- *p* = 0.5 (assumed proportion)
- *e* = 0.05 (margin of error)

Accordingly, 369 surveys were distributed to eligible healthcare providers. A total of 147 surveys were returned (39.8% of those distributed). Of these, 12 surveys (8.2% of the returned questionnaires) were excluded due to missing or incomplete data, leaving 135 fully completed surveys for analysis. This represents an effective response rate of 36.6% of the originally distributed questionnaires. The lower-than-expected participation was mainly due to heavy clinical workloads, competing time demands, and shift schedules that limited staff availability.

### 2.5 Data collection tool and measures

Data on job satisfaction were collected using a structured questionnaire adapted from Deshmukh et al. (2023). The instrument was distributed electronically via Google Forms. Links to the survey were first shared with the medical directors of King Khalid University Hospital (KKUH) and Prince Sultan Military Medical City (PSMMC), who then disseminated them to departmental heads for circulation among their staff.

The questionnaire consists of 36 items, organized into nine subscales, each representing a core domain of job satisfaction: Pay, Promotion, Supervision, Fringe Benefits, Contingent Rewards, Operating Procedures, Co-workers, Nature of Work, and Communication. Each subscale includes four items.

Participants rated each item using a six-point Likert scale, ranging from 1 (Disagree very much) to 6 (Agree very much). Several negatively worded items were reverse-coded prior to analysis to ensure consistent scoring. The total job satisfaction score ranges from 36 to 216, with higher scores indicating greater levels of satisfaction.

For analytical purposes, job satisfaction levels were categorized based on total scores as follows: dissatisfied (36–108), ambivalent (109–156), and satisfied (157–216). Each subscale captures a distinct aspect of job satisfaction. Each subscale has a possible score range of 4 to 24, with higher values reflecting greater satisfaction in that specific domain. Mean subscale scores were calculated by summing responses within each domain and dividing by the number of respondents. These scores were interpreted relative to the possible range (e.g., values closer to 24 indicate higher satisfaction). Examples of representative items include the following:

- *Pay*: “I feel I am being paid a fair amount for the work I do.”
- *Promotion*: “There is really too little chance for promotion in my job” (reverse-coded).
- *Supervision*: “My supervisor is quite competent in doing his/her job.”
- *Fringe benefits*: “The benefit package we have is equitable.”
- *Contingent rewards*: “When I do a good job, I receive the recognition for it that I should receive.”
- *Operating procedures*: “My efforts to do a good job are seldom blocked by red tape.”
- *Co-workers*: “I like the people I work with.”
- *Nature of work*: “I feel a sense of pride in doing my job.”
- *Communication*: “Communications seem good within this organization.”

The adapted survey instrument demonstrated excellent internal consistency, as reflected by a Cronbach’s alpha coefficient of 0.92, confirming its reliability for assessing job satisfaction among healthcare professionals in tertiary care settings.

### 2.6 Pilot study

A pilot test was conducted with approximately 10% of the total sample (*n* = 37) to assess the clarity and feasibility of the questionnaire. Feedback from the pilot was used to refine the survey. Participants in the pilot were excluded from the main analysis.

### 2.7 Validity and reliability

The instrument used in this study is a previously validated tool. Internal consistency reliability was confirmed with a Cronbach’s alpha coefficient of 0.92, indicating excellent reliability.

### 2.8 Data analysis

Data were analyzed using IBM SPSS Statistics version 29.0. Descriptive statistics (frequencies, percentages, means, and standard deviations) were used to summarize participants’ demographic characteristics and job satisfaction scores. The chi-square test of independence (*χ*^2^) was used to assess associations between categorical demographic variables (e.g., age group, gender, nationality, employment status, and years of experience) and job satisfaction categories (dissatisfied, ambivalent, satisfied). The one-way analysis of variance (ANOVA) was applied to compare mean satisfaction scores across occupational groups (doctors, nurses, and allied health professionals) and across subscales of job satisfaction. An independent-samples *t*-test was used where appropriate to compare mean satisfaction scores between two groups, such as male vs. female participants or Saudi vs. non-Saudi nationals. A *p*-value <0.05 was considered statistically significant.

### 2.9 Inclusion criteria

All healthcare providers working at KKUH and PSMMC during the study period who agreed to participate.

### 2.10 Exclusion criteria

Healthcare providers who were unavailable during data collection or declined to participate.

### 2.11 Ethical considerations

Ethical approval for this study was obtained from the Institutional Review Board at King Saud University (IRB Reference No. KSU-HE-25-083). Participation was voluntary, and all respondents provided informed consent prior to completing the questionnaire. The study procedures adhered to ethical standards to ensure confidentiality, privacy, and the rights of all participants. Data were collected anonymously during February and March 2025.

## 3 Results

### 3.1 Demographic characteristics

Table 1 presents the demographic characteristics of the study participants (*N* = 135). Respondents varied in age, gender, years of professional experience, employment status, nationality, and occupational role.

**Table 1:** Demographic characteristics of respondents (*N* = 135).

Characteristics	*n*	%
Age (years)		
21–30	38	28.1%
31–40	58	42.9%
41–50	19	14.1%
50 and above	20	14.9%
Gender		
Male	58	42.9%
Female	77	57.1%
Experience		
Below 2 years	19	14.1%
2–5 years	38	28.1%
Above 5 years	78	57.8%
Employment status		
Regular	76	56.3%
Contractual	59	43.7%
Nationality		
Saudi	96	71.1%
Non-Saudi	39	29.9%
Occupation		
Doctors	53	39.2%
Nurses	43	31.9%
Allied health professionals	39	28.9%

The majority of participants were aged 31–40 years 58 (42.9%), followed by those aged 21–30 years 38 (28.1%). Participants aged 50 years and above comprised 20 (14.9%), while those aged 41–50 represented 19 (14.1%). The age distribution suggests a predominance of early- to mid-career professionals in the healthcare workforce.

Females constituted the majority of respondents 77 (57.1%), while males accounted for 58 (42.9%), reflecting the gendered distribution of roles, particularly in nursing.

More than half of the participants 78 (57.8%) reported over 5 years of professional experience, followed by 38 (28.1%) with 2–5 years, and 19 (14.1%) with less than 2 years.

In terms of employment status, 76 (56.3%) held regular positions, while 59 (43.7%) were contractual staff.

Most respondents were Saudi nationals 96 (71.1%), with the remaining 39 (28.9%) being non-Saudi.

Regarding occupational roles, doctors represented the largest group 53 (39.2%), followed by nurses 43 (31.9%) and allied health professionals 39 (28.9%).

### 3.2 Overall job satisfaction

**Figure 1:**
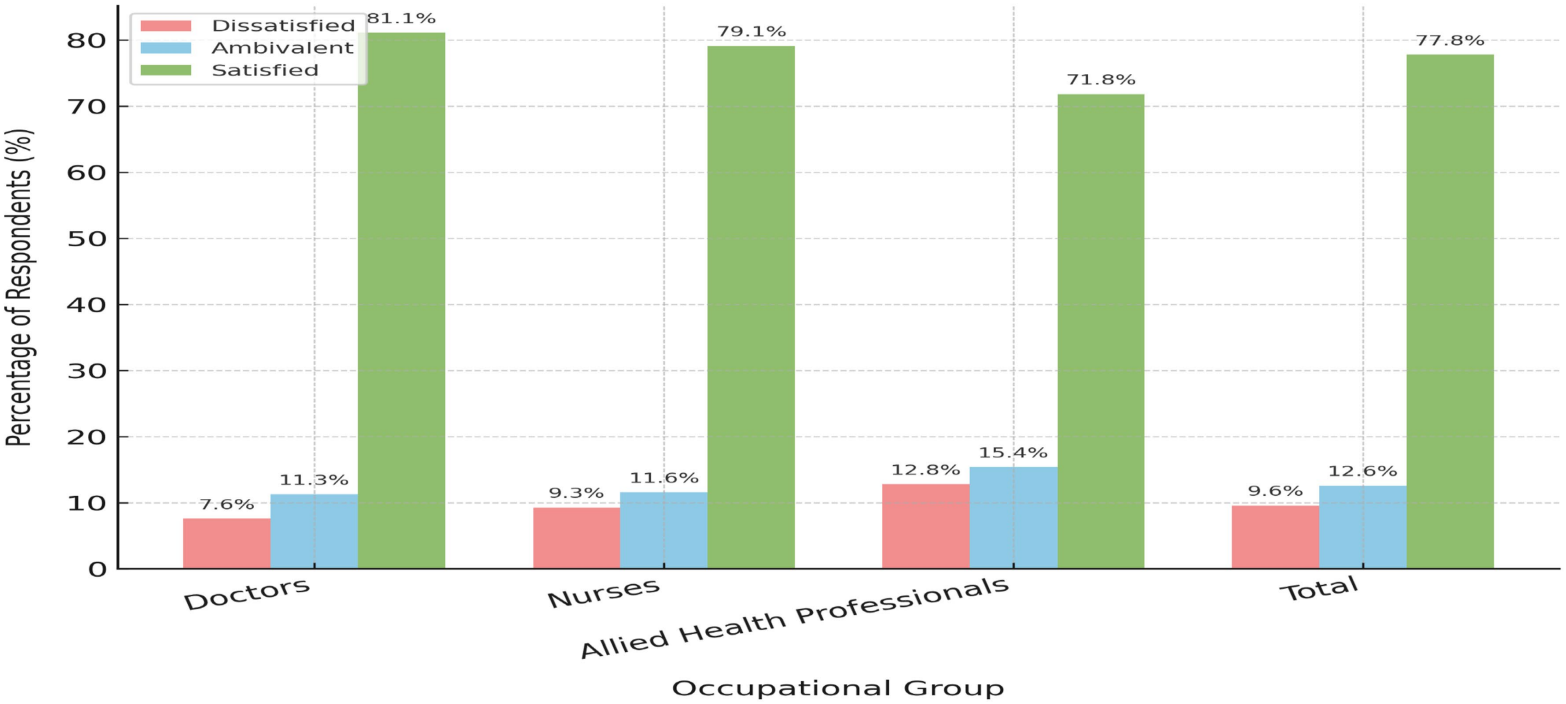
Distribution of job satisfaction levels among healthcare providers by occupational group.

As shown in Figure 1, most participants (*n =* 105, 77.8%) reported being satisfied with their jobs. A smaller proportion were ambivalent (*n =* 17, 12.6%), while 13 participants (9.6%) reported dissatisfaction.

Across occupational groups, job satisfaction appeared highest among doctors (81.1%), followed by nurses (79.1%) and allied health professionals (71.8%). Dissatisfaction was most common among allied health professionals (12.8%), compared with nurses (9.3%) and doctors (7.6%). These variations, however, were not statistically significant (χ^2^ = 1.87, *p* = 0.759, df = 4).

The mean satisfaction scores reflected a similar trend: doctors reported the highest average score (173.4 ± 21.2), followed by nurses (169.8 ± 18.7) and allied health professionals (165.2 ± 20.5). Although these figures suggest possible differences between groups, the observed variations did not reach statistical significance.

### 3.3 Job satisfaction by demographic characteristics

Table 2 shows the distribution of job satisfaction across demographic variables.

**Table 2:** Distribution of job satisfaction according to different demographic characteristics.

Characteristics	Dissatisfied 36–108		Ambivalent 109–156		Satisfied 157–216		DF1	*χ* ^2^	*p*-value
	Freq.	%	Freq.	%	Freq.	%			
Age (years)									
21–30 (*n* = 38)	4	10.1	6	15.9	28	73.7			
31–40 (*n* = 58)	2	3.4	7	12.1	49	84.5	3	4.57	0.0138
41–50 (*n* = 19)	3	15.7	4	21.1	12	63.2			
50 and above (*n* = 20)	3	15	3	15	14	70			
Gender									
Male (*n* = 59)	6	10.3	5	8.5	48	81.2			
Female (*n* = 77)	2	2.6	4	5.2	71	92.2	1	5.44	0.0123
Experience									
Below 2 years (*n* = 19)	2	10.5	4	21.1	13	68.4			
2–5 years (*n* = 38)	2	5.3	1	2.6	35	92.1			
Above 5 years (*n* = 78)	3	3.8	4	5.1	71	91	2	3.87	0.0096
Employment status									
Regular (*n* = 76)	1	1.4	3	3.9	72	94.7	1	6.21	0.0126
Contractual (*n* = 59)	2	3.4	5	8.5	52	88.1			
Nationality									
Saudi (*n* = 96)	3	3.1	4	4.2	89	94.7	1	7.13	0.0173
Non-Saudi (*n* = 39)	3	7.7	2	5.1	34	87.2			

*χ*^2^, Chi-square test of independence; df, degrees of freedom; *p* < 0.05 considered statistically significant.

Participants aged 31–40 exhibited the highest satisfaction (84.5%), while satisfaction was lowest among those aged 41–50 (63.2%).

Female respondents reported significantly higher satisfaction (92.2%) compared to males (81.2%) (χ^2^ = 5.44, *p* = 0.0123).

Participants with more than 5 years of experience had high satisfaction rates (91%), as did those with 2–5 years (92.1%). Satisfaction was lowest among those with less than 2 years of experience (68.4%) (χ^2^ = 3.87, *p* = 0.0096).

Regular employees reported higher satisfaction (94.7%) compared to contractual staff (88.1%) (χ^2^ = 6.21, *p* = 0.0126).

Saudi nationals reported higher satisfaction (94.7%) than non-Saudis (87.2%) (χ^2^ = 7.13, *p* = 0.0173).

### 3.4 Subscale-specific job satisfaction

Among the nine subscales assessed, contingent rewards recorded the highest satisfaction level, with 96.3% of participants reporting favorable perceptions. This was followed by fringe benefits (95.6%), supervision (94.8%), co-worker relationships (94.8 percent), and operating procedures (94.1%). High satisfaction levels were also noted for promotional opportunities and communication, both at 93.3%.

Slightly lower satisfaction was reported for nature of work (91.9%) and pay (90.4%), with pay representing the lowest rated domain (Table 3).

**Table 3:** Job satisfaction for the four-item subscales for all participants.

Subscales	Satisfied		Dissatisfied		Ambivalent	
	*Freq.*	*%*	*Freq.*	*%*	*Freq.*	*%*
Pay	122	90.4	5	3.7	8	5.9
Promotion	126	93.3	4	3	5	3.7
Supervision	128	94.8	3	2.2	4	3
Fringe benefits	129	95.6	2	1.5	4	3
Contingent rewards	130	96.3	3	2.2	2	1.5
Operating procedures	127	94.1	5	3.7	3	2.2
Co-workers	128	94.8	3	2.2	4	3
Nature of work	124	91.9	5	3.7	6	4.4
Communication	126	93.3	3	2.2	6	4.4

Overall, the results reflect a strong level of satisfaction across all job dimensions, indicating that healthcare providers generally perceive their work environment in a positive manner.

### 3.5 Comparison across occupational groups

Dissatisfaction levels varied notably across professional groups. Among doctors, the highest dissatisfaction was observed in relation to co-worker relationships (47.8%), operating procedures (43.3%), and pay (42.6%). Nurses, on the other hand, reported the greatest dissatisfaction with communication (88.9%), followed by pay (75.4%) and promotion opportunities (72.2%). Allied health professionals expressed the highest dissatisfaction with contingent rewards (93.1%), promotion prospects (88.1%), and pay (82%). Communication emerged as a significant concern, particularly among nurses and allied health professionals, who reported low satisfaction rates of 11.1 and 17.5%, respectively.

### 3.6 Differences in satisfaction across occupational groups

Mean satisfaction scores across the nine subscales (possible range: 4–24 per subscale) varied by occupational group (Table 4). Allied health professionals reported the highest satisfaction with the nature of their work (mean = 20.00), followed by nurses (mean = 18.00) and doctors (mean = 17.98). In contrast, communication received the lowest mean score among allied health professionals (mean = 5.64), while doctors (mean = 16.32) and nurses (mean = 14.54) reported higher values within the same domain.

**Table 4:** Comparison of mean satisfaction score among all three groups (for the four-item subscales).

Subscales	Mean scores		
	Doctors	Nurses	Allied health professionals
Pay	15.65	12.45	14.98
Promotion	16.76	13.93	15.33
Supervision	12.63	16.43	14.83
Fringe benefits	17.34	16.43	11.54
Contingent rewards	17.38	16.43	12.84
Operating procedures	15.67	14.65	13.63
Co-workers	13.84	14.22	13.55
Nature of work	17.98	18	20
Communication	16.32	14.54	5.64

Satisfaction with contingent rewards and fringe benefits was highest among doctors (means = 17.38 and 17.34, respectively), with nurses showing similar levels of satisfaction (means = 16.43), while allied health professionals reported lower scores (means = 12.84 and 11.54).

Doctors also reported the highest satisfaction with promotion (mean = 16.76) and pay (mean = 15.65), while nurses scored highest for supervision (mean = 16.43). Allied health professionals showed comparatively lower satisfaction across most domains, except for nature of work. The mean scores for co-worker relationships and operating procedures were relatively consistent across groups, though slightly higher for doctors.

Overall, these findings highlight key areas of strength (such as meaningful work and peer relationships), while identifying communication and compensation as areas where satisfaction was lower, particularly among allied health professionals.

## 4 Discussion

This study aimed to assess job satisfaction among healthcare providers in two tertiary government hospitals in Riyadh, Saudi Arabia, and to examine differences across professional roles and satisfaction domains. The demographic profile of the respondents (*N* = 135) provides insight into the diverse characteristics of the healthcare workforce, including age, gender, experience, and professional roles, all of which influence satisfaction and service delivery outcomes.

The majority of healthcare workers were between 31 and 40 years old (42.9%), followed by those aged 21–30 (28.1%). This aligns with previous findings indicating a predominance of early to mid-career professionals in Saudi Arabia’s healthcare system (Al-Haroon and Al-Qahtani, 2020). While a younger workforce may be more adaptable to new technologies and workflows, they may also be more susceptible to burnout if institutional support is lacking. The female predominance (57.1%) reflects national and global trends, particularly in the nursing profession (Halawani et al., 2021) and suggests a need for institutional policies that support work-life balance, maternity leave, and career development tailored to women.

A significant proportion of participants (57.8%) had over 5 years of experience, indicating a relatively seasoned workforce. Research suggests that more experienced healthcare providers often report higher job satisfaction and lower turnover intentions [4]. Conversely, respondents with less than 2 years of experience (14.1%) may benefit from increased supervision and structured mentorship to enhance retention.

Permanent employment was more common (56.3%) than contractual roles (43.7%), a balance that has been linked to job satisfaction in the literature. Contractual staff often experience lower job security, fewer benefits, and reduced institutional integration, which may negatively influence morale (Wang et al., 2020).

The predominance of Saudi nationals (71.1%) supports ongoing localization efforts in line with Vision 2030. Compared to earlier reports (Ramli, 2019), this indicates increasing success in nationalizing the healthcare workforce in government hospitals. The professional distribution among doctors (39.2%), nurses (31.9%), and allied health professionals (28.9%) was relatively balanced, offering a comprehensive perspective on job satisfaction across occupational groups (Raoush, 2023).

Overall, 77.8% of healthcare providers reported being satisfied with their jobs. Doctors showed the highest satisfaction (81.1%), followed by nurses (79.1%) and allied health professionals (71.8%). These results contrast with earlier findings where support staff typically reported higher satisfaction (Raoush, 2023), potentially due to different stressors and organizational dynamics. In this study, the lower satisfaction among allied health professionals may reflect unmet expectations or fewer opportunities for advancement (Wang et al., 2020).

Although doctors had the highest mean satisfaction score (173.4 ± 21.2), this difference was not statistically significant across occupations (*p* = 0.759), suggesting a generally uniform satisfaction level or a sample size insufficient to detect variation. This aligns with the notion that multiple job satisfaction determinants, such as autonomy and compensation, may be consistently distributed (Alghamdi and Alotaibi, 2023).

Age-wise, the 31–40 group had the highest satisfaction (84.5%), with a gradual decline in older age groups. This trend may relate to career plateau, increased responsibilities, or burnout (Wang et al., 2020). Female participants reported higher satisfaction (92.2%) than males (81.2%). While this finding aligns with evidence that women in caregiving roles often derive intrinsic satisfaction from their work (Al-Dosari et al., 2023), it should be interpreted with cultural and institutional sensitivity. In the Saudi context, workplace policies and initiatives that support women’s participation in healthcare, including maternity benefits, flexible scheduling, and career development programs, may contribute to higher reported satisfaction. At the same time, gender-based differences in satisfaction are not consistently observed in the literature (Ahmed and El-Sayed, 2023), suggesting that these results may also reflect contextual factors such as workplace culture and role expectations. Satisfaction increased with experience, reaching 91% among those with over 5 years in service. This aligns with evidence suggesting that workplace familiarity and professional competence enhance job satisfaction (Raoush, 2023). Similarly, regular employees reported higher satisfaction than contractual staff, likely due to greater job security and institutional support (Almalki et al., 2012).

Saudi nationals (94.7%) reported higher satisfaction than non-Saudis (87.2%). Although the gap is modest, it likely reflects the influence of workforce nationalization initiatives under Vision 2030, which emphasize employment security, career pathways, and institutional integration for Saudi healthcare workers. In contrast, non-Saudi staff may encounter challenges such as contract-based employment, limited promotion opportunities, or differential access to benefits, which could affect their satisfaction. Nonetheless, the relatively high satisfaction levels among expatriates (87.2%) suggest that the work environment remains broadly inclusive and supportive (Albugami et al., 2024).

High satisfaction was reported in subscales including contingent rewards (96.3%), fringe benefits (95.6%), and supervision (94.8%), echoing findings that institutional support and non-monetary incentives are vital to job satisfaction (Al-Dosari et al., 2023). Fringe benefits and contingent rewards had the highest average scores, reinforcing their importance (Ahmed and El-Sayed, 2023). Despite this, pay had the lowest mean score (1.86), even though 90.4% of participants reported being satisfied. This apparent discrepancy may reflect perceived inequities, particularly when compared to regional or international standards (Alkanfari et al., 2022; Moafa, 2023).

The “nature of work” subscale showed a high satisfaction frequency (91.9%) but a lower mean score (1.88), suggesting workload stress or ambiguity in job roles. These findings mirror reports from similar contexts, where intrinsic motivation is high despite operational pressures (Sharma et al., 2020).

In contrast to international findings of widespread dissatisfaction with operating procedures (Omar et al., 2016), the current study recorded 94.1% satisfaction in this domain, likely reflecting improvements from recent healthcare reforms. Communication and promotion were also well-rated (93.3%), which differs from studies in lower-income settings that cite these as areas of concern (Asegid et al., 2014).

Pay dissatisfaction was particularly high among nurses (75.4%) and allied health professionals (82%) versus doctors (42.6%). This pattern suggests a tiered compensation structure favoring physicians, contributing to lower morale among mid-level staff (Asegid et al., 2014).

Support staff also reported low satisfaction in contingent rewards (6.9%) and promotion opportunities (11.9%), indicating a need for recognition and career development programs (Raoush, 2023).

Interestingly, nurses reported the highest supervision satisfaction, whereas doctors were least satisfied in this area, possibly reflecting autonomy preferences or differing managerial styles. The “nature of work” received the highest mean scores across groups, showing that despite institutional challenges, healthcare providers find intrinsic value in their work (Khan et al., 2019). Strong peer support was also evident, with consistently high satisfaction in the “co-worker” subscale.

### 4.1 Implications

The findings of this study have important implications for healthcare workforce management in tertiary care settings. Notable disparities in job satisfaction across professional roles, employment types, and nationalities suggest that a one-size-fits-all approach to human resource policies may not be effective. Specifically, contractual and non-Saudi employees, as well as allied health professionals, reported comparatively lower satisfaction levels, highlighting the need for more equitable and inclusive institutional practices (Alkanfari et al., 2022).

Among the most reported areas of dissatisfaction were pay and opportunities for promotion. Hospitals should critically evaluate existing compensation structures, particularly for mid-level providers such as nurses and allied health staff and ensure that transparent and fair career advancement pathways are in place (Alharthi et al., 2023). Enhanced supervision, peer support, and recognition initiatives may also play a key role in mitigating emotional exhaustion and fostering stronger organizational commitment (Alshaibani et al., 2024).

Moreover, in tertiary healthcare institutions where clinical service and education intersect, job satisfaction has a direct bearing on the learning environment. Medical educators not only deliver patient care but also serve as mentors and role models for trainees. Dissatisfaction in areas such as communication, recognition, and institutional support may impair the quality of teaching and diminish student engagement (Reeves et al., 2017). Ensuring that educators receive adequate institutional support, professional development opportunities, and recognition for their dual roles is essential for sustaining high standards in both care and education.

### 4.2 Implications for organizational psychology in healthcare settings

This study offers valuable insights into how organizational factors influence job satisfaction within healthcare settings, especially in demanding environments such as tertiary hospitals. Nurse managers and healthcare leaders play an essential role in shaping the psychological work climate. Their actions can either support or weaken staff well-being, motivation, and commitment.

Although many healthcare providers reported moderate satisfaction, nurses and allied health professionals expressed lower satisfaction in important areas such as compensation, opportunities for promotion, and recognition. These findings highlight the need for workplace practices that promote fairness, emotional safety, and a sense of belonging.

Supportive and inclusive leadership practices can help build a more positive and resilient organizational culture. Efforts to ensure fair promotion pathways, meaningful recognition, and adequate compensation are not just administrative concerns; they directly influence how staff feel about their work and their value within the organization.

Providing mentorship and professional development is especially helpful for staff early in their careers or those with responsibilities in both clinical and teaching roles. Encouraging their growth builds confidence, strengthens skills, and promotes long-term engagement.

It is also important to consider the experiences of contractual and non-national staff, who may face challenges related to job security or organizational integration. Policies that include all employee groups and value diversity help improve team collaboration and reduce feelings of exclusion.

By prioritizing leadership development and fostering a psychologically supportive environment, healthcare organizations can improve job satisfaction, enhance retention, and support better patient outcomes. These practices also align with global goals for good health, decent work, and reduced inequalities.

### 4.3 Recommendations

For Hospital Administrations:

1. Review pay scales for nurses and allied health professionals.
2. Improve communication systems, especially for nurses and support staff.
3. Establish clear, equitable promotion policies.
4. Enhance recognition and reward programs.
5. Support the integration and professional development of contractual staff.
6. Foster inclusive leadership to promote equity.

For the Ministry of Health:

1. Standardize compensation policies across institutions.
2. Create initiatives for professional growth under Vision 2030.
3. Promote career security through nationalization and fair employment policies.

### 4.4 Limitations and future directions

Several limitations should be acknowledged when interpreting the findings of this study. First, the cross-sectional design precludes any conclusions regarding causal relationships between job satisfaction and demographic or occupational variables. Longitudinal research is required to better capture changes in satisfaction levels over time and in response to policy or organizational reforms.

Second, the relatively small sample size (*n =* 135) and moderate response rate may have reduced the statistical power of the analyses and limited the representativeness of the findings. Future studies with larger, more diverse samples across multiple institutions would enhance the generalizability of results.

Third, the study was conducted in only two tertiary care government hospitals in Riyadh, which may restrict the applicability of the findings to other healthcare settings, regions, or organizational structures within Saudi Arabia. Broader sampling across different healthcare systems is recommended.

Fourth, the reliance on self-reported questionnaires introduces the possibility of response bias. Despite assurances of anonymity, participants may have been inclined to provide socially desirable responses, potentially inflating reported levels of satisfaction. Incorporating qualitative methods or triangulating survey data with objective measures in future studies could mitigate this limitation.

Finally, while the structured quantitative approach offered a broad assessment of job satisfaction across multiple domains, it may not fully capture the nuanced and subjective experiences of healthcare providers. Mixed-methods designs are recommended to generate a more comprehensive understanding by integrating numerical trends with qualitative insights.

## 5 Conclusion

The study underscores the overall high job satisfaction among healthcare providers in Riyadh’s tertiary government hospitals, while also highlighting specific areas of concern across different occupational groups. Addressing these variations through tailored policies and organizational interventions is essential to promoting a motivated, efficient, and education-focused healthcare workforce in Saudi Arabia.

## Data Availability

The raw data supporting the conclusions of this article will be made available by the authors, without undue reservation.
